# Asthma and COPD: Comparison with International Guidelines and Medication Adherence in Belgium

**DOI:** 10.3390/ph16071030

**Published:** 2023-07-20

**Authors:** Natacha Biset, Mélanie Lelubre, Stéphanie Pochet, Carine De Vriese

**Affiliations:** Department of Pharmacotherapy and Pharmaceutics, Faculty of Pharmacy, Université Libre de Bruxelles (ULB), 1050 Brussels, Belgium; natacha.biset@ulb.be (N.B.);

**Keywords:** medication adherence, inhaled treatment, asthma, COPD, new medicines service

## Abstract

Asthma and chronic obstructive pulmonary disease (COPD) are major chronic conditions. It is possible to limit their impact by controlling symptoms, which limits exacerbations and worsening of the disease, by choosing the appropriate treatment and ensuring that the patient adheres to it. The main purpose of this study was to assess medication adherence and persistence with inhaled medications for chronic treatment of asthma and COPD, as well as to evaluate the factors influencing this adherence. Medication adherence was measured from January 2013 to December 2016 using continuous multiple-interval measures of medication availability (CMA). Persistence was evaluated by treatment episodes (TE). We analyzed the influence of different factors on CMA such as sex, age, type of device, and the realization of the “new medicines service” (NMS), introduced in Belgium in October 2013 to support patients in adhering to their treatment. We also analyzed the consumption of these inhaled medications within the Belgian population and compared them with the Global Initiative for Asthma (GINA) and the Global Initiative for Chronic Obstructive Lung Disease (GOLD) recommendations. Medication adherence varied greatly between the different pharmacological classes: inhaled corticosteroids (ICS) alone or in combination with long-acting beta agonists (LABA) had the lowest medication adherence and persistence, while adherence was highest for the long-acting muscarinic antagonists (LAMA) and LABA/LAMA associations. The NMS seemed to have a positive impact on medication adherence, although few patients completed the two guidance interviews offered by the service. In addition, only a minority of the targeted patients took advantage of this new service.

## 1. Introduction

Asthma and chronic obstructive pulmonary disease (COPD) are major chronic conditions. In 2019, asthma affected about 6% of the Belgian population over 15 years old, while this rate was 4% for COPD [1]. According to the World Health Organization (WHO), asthma is one of the most common chronic diseases in children and COPD is the third leading cause of death in the world [2,3]. It is possible to limit the impact of these conditions by controlling symptoms, which limits exacerbations and worsening of the disease. One of the keys to achieving this is the appropriate choice of treatment. Guidelines issued by the Global Initiative for Asthma (GINA) and the Global Initiative for Chronic Obstructive Lung Disease (GOLD) were developed to help health professionals in this choice and are based on recent scientific literature. These guidelines have become a reference for the management of these pathologies [4,5]. However, adherence to these guidelines seems to be suboptimal despite their proven added value. This may be linked to several barriers, including healthcare practitioners’ lack of familiarity with the guidelines, lack of time, or the difficulty in applying the guidelines in daily practice [6,7,8].

Another key to the good management of these diseases is proper intake of the treatment [9,10]. However, poor medication adherence in chronic treatment is very common. In 2003, the WHO estimated that only 50% of patients with chronic diseases were adhering to their treatment. This proportion of adherent patients was much lower for patients in developing countries [11]. Since this observation was made, many additional studies have shown that adherence in chronic diseases is not optimal [12,13,14].

Medication adherence is defined by three components: initiation, implementation, and persistence. The patient initiates the treatment by coming to the pharmacy to pick up the treatment that has been prescribed. Then, if the patient follows the treatment continuously according to the prescription, this is called “implementation”. Finally, persistence is measured by the amount of time that has elapsed between initiation and discontinuation, namely, the moment when the patient stops the treatment [15].

Non-adherence to medications seems to delay or compromise patient recovery, making the illness more severe and resulting in an increase in hospitalizations and healthcare expenses. Among patients with COPD, non-adherence is also associated with a decrease in the quality of life [16]. Improving medication adherence is therefore important for asthma and COPD treatments. Beyond medication adherence, a correct inhalation technique is essential for good delivery of the drug. Good use of the device allows for better pulmonary function and better health [17,18].

Since October 2013, a new service has been developed and implemented in Belgium. This service is dispensed by pharmacists and is entitled the “new medicines service” (NMS). It is available to all asthma patients receiving inhaled corticosteroids (ICS) for the first time or who have not received ICS for a year. It may also be prescribed to patients outside these groups by a physician. The service consists of two interviews during which the disease, its treatments, and their proper use are explained, as well as the importance of medication adherence [19,20].

The main purpose of this study was to assess medication adherence and persistence with inhaled medications for the chronic treatment of asthma and COPD as well as to evaluate the factors influencing this adherence. The influencing factors studied were sex, age, type of device and the new service proposed by pharmacists (NMS) for ICS. We also analyzed the consumption of these medications within the Belgian population and compared them with the GINA and GOLD recommendations.

## 2. Results

### 2.1. Population, Most Prescribed Drugs, and Main Prescribers

The initial database contained almost fourteen million deliveries of the studied drugs to almost three million patients. The final population studied is detailed in Table 1.

Each pharmacological class studied contained at least three molecules on the market in Belgium at the time of the study. Table 2 shows the three most-prescribed molecules for each pharmacological class.

For all pharmacological classes, the main prescribers for these drugs were general practitioners. These were followed by pediatricians, specialists in respiratory medicine or specialists in internal medicine, depending on the pharmacological class (Table 3).

### 2.2. Medication Adherence

The long-acting beta agonists and long-acting muscarinic antagonists (LABA-LAMA) combination were the pharmacological classes with the highest medication adherence, with 75.66% of adherent patients (mean continuous multiple-interval measures of medication availability (CMA) = 0.872). LAMA and LABA alone were next, with 62.83% and 50.01% of adherent patients, respectively. Medication adherence appeared to decrease for ICS, whether in combination with LABA or alone. Indeed, only 17.56% of patients adhered to their LABA-ICS treatment and this percentage decreased to 7.98% for ICS alone (Table 4).

#### 2.2.1. Influence of Sex, Type of Device and Age

Women who received ICS were more likely to adhere to their treatment than men. The opposite was the case for all other pharmacological classes, where men tended to be more adherent than women. All differences were significant with *p* < 0.001 (Mann–Whitney test) except for LABA-LAMA, for which *p* = 0.339 (Table 5).

Concerning the type of device, for ICS, LABA and LABA-LAMA associations, patients using a dry powder inhaler (DPI) were significantly more adherent than those using a pressurized bottle (pMDI). We observed the opposite trend for LAMA and LABA-ICS. All differences were statistically significant, with *p* < 0.001 (Table 5).

Finally, regarding age, our results suggested that medication adherence seemed to increase significantly with age for all pharmacological classes (ICS: r_s_ = 0.365; LABA: r_s_ = 0.199; LAMA: r_s_ = 0.071; LABA-ICS: r_s_ = 0.211; LABA-LAMA: r_s_ = 0.091; *p* < 0.001)

#### 2.2.2. Influence of the New Medicines Service

**Figure 1:**
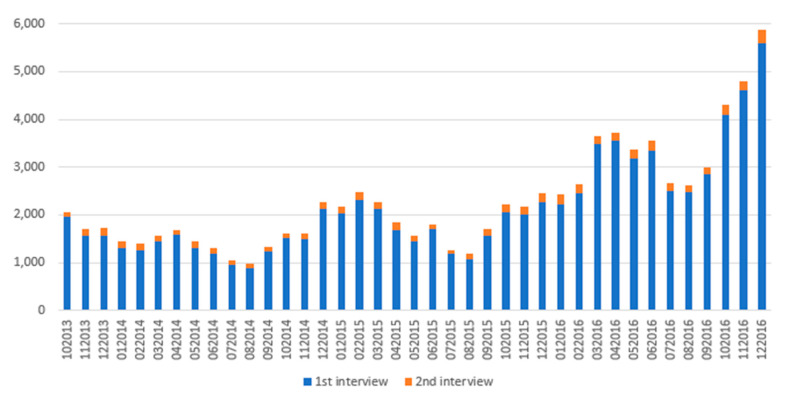
Number of interviews under the NMS performed per month during the study period since its implementation in Belgium in October 2013.

The NMS was implemented in Belgium in October 2013. Figure 1 shows the evolution over time of the number of interviews conducted during the study period.

The group of patients who conducted one or two interviews seemed to have a significantly higher mean CMA than those who did not conduct any interview (Table 6).

Patients who completed the second interview, and therefore both treatment-management interviews, seemed to be significantly more adherent than those who completed only the first interview (Table 7).

#### 2.2.3. Persistence

According to our results, for LABA and LABA-LAMA associations, the proportion of patients adhering to their treatment seemed to decrease between the first and second treatment episode (TE). For LAMA and LABA-ICS, this percentage seemed to increase for the second episode while for ICS, it remained stable. Between the second and subsequent episodes, the percentage of adherent patients increased for all pharmacological classes (Table 8).

## 3. Discussion

### 3.1. Population and Comparaison with GINA and GOLD Guidelines

The most common pharmacological class delivered to treat asthma and COPD was the LABA-ICS combination, followed by ICS alone. These two classes represented almost 80% of deliveries in Belgium for such treatment over the period studied and were received by 86.4% of the patients. However, we noted that these two classes were not delivered to the same population. ICS were mostly delivered to a younger population than LABA-ICS combinations (mean age: 31 vs. 53 years old, respectively). This appeared to be consistent with the Global Initiative for Asthma (GINA) and the Global Initiative for Chronic Obstructive Lung Disease (GOLD) management guidelines at the time of the study. For the treatment of COPD, ICS alone were a possibility but their combination with LABA seemed to be more effective, while low-dose ICS could be considered in the early stages of asthma treatment [5,21]. However, we noticed a significant delivery of ICS to young children in our database with a median age of 14 years for this pharmacological class (Table 1). This could be explained by GINA guidelines at the time of the study recommending the use of moderate doses of ICS for children between 6 and 11 years old, with an as-needed short-acting beta agonist (SABA) as a reliever for the second and third step of the treatment. This is in contrast with the recommendation to use of a LABA-ICS combination to initiate the third step of treatment in patients over 12 years old. For children under the age of five with asthma, daily low doses of ICS were recommended for the second step of the treatment. It has already been shown that young children (2–6 years) were more likely to receive asthma medications such as ICS than older children [22]. However, for these young children, the diagnosis of asthma is difficult to establish because of the non-specificity of the symptoms and the lack of tests available for these symptoms [23]. Indeed, wheezing is not always synonymous with asthma because children at this age are very likely to have viral-induced wheezing [4]. In addition, ICS can be used off-label in young children to treat, for example, acute bronchitis or upper-respiratory-tract conditions [24].

As mentioned above, for patients over 12 years old with asthma, LABA-ICS combinations were the preferred choice of control for the third step of treatment. For COPD, the treatment must be adapted for each patient because it will depend on the severity of symptoms and limitation of airflow related to exacerbations, on the presence of respiratory failure, on comorbidities, or on the general health of the patient. According to GOLD guidelines in progress at the time of the study, the LABA-ICS association was more effective at improving lung function and patient health, as well as in reducing exacerbations, than either of the individual components [5]. This may also explain the higher average patient age for LABA-ICS than for ICS, alone as this combination was often used to treat COPD patients or for asthma patients in more severe stages of the disease. The use of LABA and LAMA was also recommended to treat COPD. The pharmacological class used will depend on the patient’s response to treatment. The combination of drugs will also improve their efficacy and reduce the risk of side effects. Looking at our results (Table 1), it seemed that LAMA were prescribed more often than LABA. LABA-LAMA combinations were prescribed less often, although they would be preferable to LABA-ICS combinations because they would increase lung capacity while significantly decreasing the risk of pneumonia and exacerbations [25]. Regarding the use of bronchodilators in asthma, LABA and LAMA monotherapy was not recommended for treatments but recommended for use as an add-on treatment for more severe forms of the disease. Therefore, according to the guidelines, LABA, LAMA and their combinations were mostly delivered in the context of COPD [5,21]. This could explain the population characteristics for these three pharmacological classes in our study. Indeed, the average age ranged from 64 to 68 years for these three pharmacological classes, compared with 31 to 53 years for ICS and LABA-ICS.

Concerning the most-dispensed molecules for each class (Table 2), for ICS, only three molecules were available in Belgium for inhalation. The GINA and GOLD guidelines in progress at the time of the study period did not recommend one molecule over another. Tiotropium was the most delivered LAMA in our study (91.38%). This preference was probably related to the GOLD guidelines emphasizing it because it seemed to improve pulmonary rehabilitation. Regarding LABA-ICS combinations, mainly two were delivered: salmeterol and fluticasone (38.98%) and formoterol and budesonide (36.09%). In third place were formoterol and beclomethasone (17.51%). According to the GINA guidelines, it was preferable to use a combination of ICS with formoterol for patients at risk because this would reduce the risk of exacerbations and would control asthma at lower doses than other combinations or ICS alone [21]. However, the GOLD guidelines did not recommend one combination over another [5]. Finally, to explain the choice of one molecule over another, some authors suggested that the content of doctors’ prescriptions may be influenced by the marketing of pharmaceutical companies [26].

For all pharmacological classes, drugs were mainly dispensed by general practitioners. GPs accounted for 34.5% of physicians in Belgium, while 1.4% of physicians were specialized in respiratory medicine [27]. Therefore, in view of the Belgian healthcare system, we assume that patients visited a specialist in respiratory medicine at the beginning of their illness for diagnosis and initiation of treatment. It is likely that the patient then went to the GP for regular check-ups and prescription renewals for the treatment established by the specialist. According to this hypothesis, the patient would go more often to their GP than to the specialist in respiratory medicine and the GP would be the main prescriber of the treatment. The specialist would be consulted for annual or occasional check-ups, in addition to the initial consultation. This seemed to be consistent with our results showing that specialists in respiratory medicine were the second-largest prescribers except for ICS. Indeed, we noticed that ICS are often prescribed by pediatricians, and therefore prescribed for young children. As mentioned earlier, the median age of the ICS population was 14 years.

### 3.2. Medication Adherence

Medication adherence ranged considerably from one pharmacological class to another. Adherence seemed to be the lowest for ICS, whereas it was highest for the LABA-LAMA combination. The average CMA was 0.872, and 75.66% of patients were adherent (Table 4).

Medication adherence to ICS seemed low, with an average CMA of 0.211 and 7.98% of patients being adherent. Engelkes et al., in a review about asthma, showed that relatively poor adherence to ICS was common. They showed that for children, medication adherence can vary from 0.200 to 0.339 while for adults it can vary from 0.15 to 0.54 [28]. Bidwal et al. found that medication adherence to ICS was quite low, with 8.3% of the population being adherent to ICS alone or combined with LABA [29]. Patients questioning the safety of corticosteroids, and therefore being afraid of them, could explain this low adherence [30]. Another hypothesis to explain this rather low percentage would be that patients were taking their medication but using a lower dose than the defined daily dose. This could be due to either the doctor prescribing a lower dose or the patient deciding to decrease the dose. Indeed, according to a study of asthma patients between the ages of 15 and 45, more than half of the patients decreased their steroid intake when their symptoms improved [31]. Furthermore, the population taking ICS in our study was quite young, and as our results and other studies showed, adherence tends to increase with age [29,32]. When looking at the GINA guidelines in effect at the time of the study, ICS were not involved in the initial stages of asthma treatment. However, ICS could also be used to treat other diseases than asthma such as acute bronchitis or upper-respiratory-tract conditions [24].

Concerning ICS associated with LABA, medication adherence appeared to be better than with ICS alone. We found that 17.56% of patients were adherent. This percentage was higher than the 8.3% found by Bidwal et al. [29], but it was still less than the 30% of patients adherent to LABA-ICS associations found by Averell et al. [33]. Averell et al. showed that, in patients with asthma, the mean proportion of days covered (PDC) was 0.63 [33]. It could even be higher for asthmatic patients initiating the treatment, with a mean PDC from 0.72 to 0.77 depending on the associations used [34]. In COPD patients, the mean PDC found for one specific combination of LABA-ICS was 0.38 [35]. Our results fell within this range as we obtained a mean CMA of 0.42. Higher medication adherence to LABA-ICS combinations compared to ICS alone could be explained by the prescription of these combinations to patients with more advanced disease. If the symptoms were more present, the patient would be more motivated to follow the treatment and consequently would tend to be more adherent [29]. In asthma, Barnes et al. showed that patients with a milder form of the disease were less likely to be adherent [36]. Furthermore, it has been shown that adherence to treatment was poorer when the treatment did not have an immediate effect on the symptoms [18]. This could also explain why medication adherence in LABA-ICS associations tended to be higher than ICS alone.

For LAMA in monotherapy, medication adherence appeared to be quite high, with a mean CMA of 0.797 and 62.83% of adherent patients. Good medication adherence for COPD patients has already been demonstrated, with a mean PDC even above 1 [37]. This means that patients were taking their medication at a higher dosage than recommended. In our methodology, we chose to limit the maximum value of PDC to 1 to avoid it being pulled up by these “over-adherent” patients. Our results seemed to be in the same range as those of Bogart et al., with a mean PDC for LAMA alone ranging from 0.50 to 0.70 [38]. When LAMA were associated with LABA, our results showed that adherence was even higher than for LAMA alone, with a mean CMA of 0.872. These combinations were mainly delivered to COPD patients and their adherence tended to be better than that of asthmatic patients [39]. This could be due, as mentioned above, to the older population of COPD patients being more adherent as well as COPD patients experiencing more severe symptoms. Nevertheless, our medication adherence results were superior to those of Moretz et al., who showed that patients starting treatment with LABA-LAMA combinations had a mean PDC of 0.47 to 0.50 and therefore that only 22.7% to 28.6% of patients were adherent. They also showed that patients starting a new treatment were more adherent when taking a combination of LABA-LAMA than a combination of LABA-ICS. We observed the same trend regardless of whether treatment was initiated or continued. These authors therefore hypothesized that the LABA-LAMA combination provided better symptom control in COPD patients and limited exacerbations [40].

Another explanation for the higher medication adherence for this pharmacological class could be the dosage. Indeed, the dosage of each drug may also play a role in adherence. For asthma and COPD, it has been shown that adherence tended to be better if the medication was taken once a day instead of twice a day [41,42,43,44]. In our results, LABA-LAMA combinations had the highest proportion of adherent patients (75.66%), and almost all the drugs had to be taken once a day. Only one specialty (a combination of formoterol and aclidinium bromide) had to be taken twice a day, but it represented less than 1% of deliveries. Concerning LAMA, which was the second-highest pharmacological class in terms of the proportion of adherent patients (62.83%), we noticed that tiotropium bromide and glycopyrronium bromide, which represented more than 97% of LAMA deliveries, were once-daily drugs. For LABA, for which 50.01% of patients were adherent, the most prescribed molecule was indacaterol (54.22%), which had to be taken once a day. The other two most-prescribed molecules, formoterol (37.21%) and salmeterol (8.57%), had to be taken twice a day. This led to a higher proportion of drugs with higher dosages in this pharmacological class, which may explain the lower adherence. Looking at LABA-ICS, the three most-dispensed combinations (92.58% of deliveries) had to be taken twice a day. Only the combination of fluticasone and vilanterol had to be taken once a day. This combination represented 5.92% of deliveries but appeared on the Belgian market during the study period, in August 2014. Since then, many other specialties with this combination have emerged in Belgium. Finally, for ICS, which was the pharmacological class with the lowest adherence, all specialties available on the Belgian market must be taken twice a day. Therefore, the influence of dosage on medication adherence appeared to be consistent with our results.

#### 3.2.1. Influence of Sex

The proportion of men is slightly higher for LABA, LAMA and their combinations (56.7–64.6%), which are prescribed more often for COPD, than ICS and LABA-ICS (47.5–48.8%). Several studies have shown the proportion COPD to be higher in men than in women, although this difference has tended to decrease, or may even no longer exist, because the sexes now have equivalent exposure to tobacco [45,46]. However, it has appeared that for the same risk exposure, women are likely to develop COPD with a more rapid progression, than men [47,48].

According to our results, women who received ICS were more likely to be adherent than men (mean CMA of 0.219 vs. 0.202, respectively). However, the proportion of adherent patients for this pharmacological class remained very low, with 8.69% of women having a CMA ≥ 0.8. The opposite was the case for all other pharmacological classes, where men seemed to be more adherent than women. This was statistically significant for LAMA, LABA and LABA-ICS combinations but not for the LABA-LAMA association. Schnoor et al. found that, with regard to COPD, men were more likely to be adherent than women [49]. An explanation could be that women were more likely to have more severe symptomatology than men for the same level of airway obstruction, for example having greater dyspnea and a lower body mass index. This resulted in a higher level of anxiety and depression in women with COPD than in men, resulting in a lower quality of life. A negative relationship between anxiety and depression and medication adherence has already been shown [47]. This trend has also been observed in other pathologies such as diabetes or cardiovascular diseases [50,51]. Manteuffel et al. suggested that women were more likely to be polymedicated, to experience adverse effects or to neglect self-care in favor of caring for other household members, and therefore had lower medication adherence [52]. In our results, this difference between women and men, while statistically significant, was slight and unlikely to have much impact in practice.

#### 3.2.2. Influence of the Device

We found that patients taking ICS, LABA alone or LABA in combination with LAMA were more adherent when the device was a dry powder inhaler (DPI) rather than a pressurized bottle (pMDI). For LABA alone or in combination with LAMA, more than 95% of the medications dispensed were for delivery via DPIs whereas for ICS, DPIs represented 30% of the deliveries. Regarding the latter, our results were in line with the results of Roy et al. [53]. For LAMA alone and LABA-ICS combinations, the trend was reversed, and medication adherence seemed significantly higher with a pMDI; yet most drugs delivered were DPI. Darba et al. showed that LABA-ICS combinations delivered by DPI had a negative impact on medication adherence [54]. This may be related to the fact that patients initiating LABA-ICS asthma therapy were more likely to have better asthma control with a pMDI than with a DPI [55]. However, the results found in the literature can be contradictory, and there were also differences between different devices within the same category [56]. Because of the large number of devices on the market, it was difficult to draw general conclusions about the influence of the type of device. On the other hand, it was already shown that adherence to inhaled therapy appeared to be lower than for oral medication because of the complexity of taking the treatment [57]. In addition, changing the inhalation device without consulting the patient first was not recommended, as this could harm control of the pathology [58]. In addition, switching from one type of device to another, e.g., to a cheaper generic, could also be detrimental to adherence [59]. Beyond medication adherence, a good inhalation technique is essential for good delivery of the drug. Indeed, good use of the device enables the patient to have better pulmonary function and better health [17,18]. Nevertheless, the inhalation technique did not seem to be checked often by healthcare professionals [60].

#### 3.2.3. The New Medicines Service

The NMS was first implemented in Belgium in October 2013. It was initially reserved for asthma patients who were starting treatment with ICS, which meant that they should not have received these treatments within the previous year. This NMS consists of two interviews. In the first interview, information is given about asthma, its treatments, and their proper use as well as the importance of medication adherence, which can be assessed using a five-item questionnaire called the Medication Adherence Report Scale (MARS). An assessment of the patient’s level of asthma control can also be performed using the Asthma Control Test (ACT). A second interview takes place three to six weeks after the first one. This follow-up interview aims to receive feedback from the patient on the new treatment and to discuss any problems encountered. The NMS is available based on a request from a doctor, a pharmacist or even the patient, as long as the admission criteria are met. The interview is free of charge for patients, and pharmacists receive €20/interview from the National Institute for Health and Disability Insurance (NIHDI) [19]. At the beginning of 2017, after the end of the period studied in our work, the conditions for access to the NMS were expanded and it was renamed “BUM asthme/GGG astma” (i.e., “for proper use of medication in asthma”). It can now be offered to asthma patients aged 50 and over taking ICS and whose asthma is not controlled. Asthma control is assessed using simple questions [61]. At the time of the study, the NMS was both a new set-up for patients and professionals, and the conditions of access were rather restrictive, which may explain why the number of interviews delivered was rather low. Indeed, although ICS alone and in combination were the two most-delivered pharmacological classes, to benefit from the NMS, patients were required to be new to the treatment. This therefore greatly reduced the target population.

According to our results, the interviews were mostly prescribed by GPs, specialists in respiratory medicine, or pediatricians. As mentioned above, pharmacists or patients themselves could request the NMS if the inclusion criteria were met. However, the coding system for the NMS did not allow the real source of a recommendation to be encoded. By default, the interview was coded as being prescribed by the physician who issued the prescription for ICS. It is therefore not possible to know whether the NMS were recommended more often by physicians, pharmacists, or patients themselves.

We noticed that the number of NMS interviews performed varied throughout the year (Figure 1). Indeed, during July and August, the number of NMS was at its lowest, while the maximum number seems to have been reached between December and April. Such seasonal patterns have also been demonstrated by Turi et al. for asthma medication deliveries. This pattern of more deliveries of asthma medications, including ICS, between November and May, corresponded to seasonal exacerbations of asthma [62]. As mentioned above, ICS can also be used to treat other diseases than asthma, such as acute bronchitis or upper-respiratory-tract conditions, that also have a seasonal frequency [24]. We noted that beyond this seasonal pattern, the number of interviews delivered increased over the study period. More interviews were conducted at the end of the study period than at the beginning. The NMS was implemented in October 2013 in Belgium, thus the increase in the number of interviews conducted could be related to a better awareness of patients and professionals to this new service. We also noticed that a very small proportion of patients completed the second interview. In fact, barely 7% of patients who completed the first interview also completed the second. However, patients who completed both interviews seemed significantly more adherent than those who completed only the first (Table 7). When comparing patients who had had one or both interviews, patients who attended both interviews appeared to have a higher CMA, even though a lower proportion of patients were adherent compared to those who did not have any interview (7.25% vs. 8.01%) (Table 6). These results would suggest that the NMS appeared to be effective. Even if the differences remained small, the results are encouraging. Further evaluation of the various impacts of the NMS is needed.

#### 3.2.4. Persistence

In general, our results show that adherence improved after a patient had three or more episodes. This appears to be contrary to results in the literature, which show that adherence often decreases with time and with the number of attempts. Indeed, an American study showed that while 30% of asthma patients starting LABA-ICS therapy were adherent during the first trimester, this percentage decreased to 18.8% in the second trimester and stabilized to around 12% after 18 months [33]. We noticed in our results that the average number of episodes was highest for LABA alone. This meant that patients more often had gaps of ≥90 days in their treatment. This class was closely followed by ICS and LABA-ICS. A German study of COPD patients showed that about two-thirds of patients were likely to have a 90-day gap in their first year of treatment. According to their results, persistence was worst for ICS while it was best for LAMA [63]. The same trend was observed in our results, with a mean duration of a first episode of 85 days for ICS and 461 days for LAMA. This could be related to the lower persistence of asthma patients. Indeed, they tended to discontinue treatment more quickly than COPD patients even if after one year the probability of persistence appeared to be similar [39]. In addition to the pharmacological class of medication, the inhaler may also play a role in persistence. In COPD, only 28% of patients using multiple inhalers seemed to be persistent (no gap ≥ 90 days) after 12 months [38]. This persistence improved when switching to a single inhaler for the different molecules rather than using different inhalers. This also had a positive impact on medication adherence [16].

Other factors may influence persistence; for example, young smokers with COPD seemed to be the least persistent. For asthma, it seemed that older men with comorbidities and a high-dose therapy were more persistent [64].

Increasing medication adherence to, and therefore also persistence with, asthma and COPD medications is a major concern because it has been shown to improve symptom control, decrease hospitalizations, and therefore decrease costs associated with these patients. In a Belgian study of COPD patients, the expense generated by an intervention to improve medication adherence appeared to be more than offset by the care avoided [65].

### 3.3. Strengths and Weaknesses

The main strength of our study is that the data analyzed include all deliveries made in all community pharmacies in Belgium. This enabled us to have an overview of the entire Belgian population. However, we did not have access to the deliveries made by hospitals, so we may have underestimated the adherence of some patients.

The use of the defined daily dose (DDD) to calculate medication adherence may also be a limitation. Nevertheless, this DDD was described for the main indication of the drug. In our case, it was always asthma and/or COPD. This therefore limited the possible variations in DDD related to the use of drugs for indications other than the main one, even if caution was still necessary [66,67].

The threshold of CMA ≥ 0.8 is frequently used in the literature to define whether patients are adherent or not. For some diseases where non-adherence can have very serious consequences, such as human immunodeficiency virus (HIV) infection, higher cut-off values may be used. As explained by Baumgartner et al., it is difficult to establish a threshold value for which a clinical difference will be observed. However, this 80% cut-off is the most frequently used in the literature [68].

In addition, many factors may influence adherence to therapy, such as: socioeconomic, therapy-related, condition-related, health system-related, and patient-related factors. Unfortunately, our database only allowed us to evaluate the impact of gender and age. It would be interesting for further evaluate the impact on adherence of factors such as the stage of disease, non-pharmacological treatments, and lifestyle changes.

As mentioned in the introduction, medication adherence is a process that varies over time and is defined by three components: initiation, implementation, and persistence. Based on our methodology, we were not able to assess all these components. Indeed, to be included in our database, the patient was required to have picked up the prescription at a pharmacy. We were unable to know the proportion of patients who did not initiate the treatment. According to Fischer et al., this primary non-adherence would concern 20% of patients suffering from various pathologies [69]. We must therefore be cautious when interpreting our results because, despite our very large population, we may still have a portion of the target population missing. Another known limitation of adherence measures by retrospective database analyses is the lack of information on the actual intake of the drug by the patient. Furthermore, in the case of inhaled drugs, as mentioned earlier, a good inhalation technique is essential for an optimal treatment effect. However, the NMS was developed to improve this point, among others. In addition, thanks to our large database, we were also able to assess medication adherence and persistence for all available inhaled medications for the treatment of asthma and COPD.

## 4. Materials and Methods

In Belgium, health insurance is mandatory. It covers healthcare expenses as well as compensation in case of incapacity for work. Thanks to this, all deliveries of medicines reimbursed by the compulsory health insurance from Belgian public pharmacies are included in a database: Pharmanet. This is the national health-care claims database of the NIHDI. Pharmanet provided us with a database containing community pharmacy refill data for inhaled medications used to treat asthma and COPD (R03 ATC class) from January 2013 to December 2016. The pharmacological classes concerned were: ICS (inhaled corticosteroids), LABA (long-acting beta agonists), LAMA (long-acting muscarinic antagonists) and combinations of ICS-LABA and LABA-LAMA. This database contained the following information for each delivery: a unique patient identification code (anonymized), year of birth, sex, date of delivery, product code delivered, quantity delivered and the specialty of the prescriber.

We conducted a retrospective study to evaluate medication adherence according to the continuous multiple-interval measures of medication availability (CMA) [70,71]. This CMA was calculated using AdhereR, a package in R developed to allow for better transparency and reproducibility in electronic healthcare data analysis [71]. Patients were required to have at least two deliveries of medication during the study period to evaluate medication adherence. As we did not have access to the prescribed dosage, we estimated the duration of an event according to the defined daily dose (DDD). Duration is defined as “the number of days the quantity of supplied medication would last if used as recommended” [71]. The DDD is defined by World Health Organization as “the assumed average maintenance dose per day for a drug used for its main indication in adults” [72]. Medication adherence was measured using the formula:

$$\mathrm{CMA}=\frac{\mathrm{sum}\;\mathrm{of}\;\mathrm{the}\;\mathrm{duration}\;\mathrm{of}\;\mathrm{the}\;\mathrm{medication}\;\mathrm{events},\;\mathrm{excluding}\;\mathrm{the}\;\mathrm{last}\;\mathrm{event}}{\mathrm{number}\;\mathrm{of}\;\mathrm{days}\;\mathrm{between}\;\mathrm{the}\;\mathrm{first}\;\mathrm{and}\;\mathrm{last}\;\mathrm{event}}$$

A medication event was defined as a recorded dispensing of a certain drug to a patient. The result of this calculation was capped at a maximum value of 1. Patients were considered as adherent if the CMA value was ≥0.8. To estimate persistence, we performed an analysis of medication adherence across treatment episodes (TE). An episode was considered new if a patient had a gap of ≥90 days between the moment when the supply of the last medication event was finished and the next event.

We analyzed the influence of different factors on the CMA, such as sex, age, type of device or the realization of the NMS.

Statistical analyses were performed using both R studio (version 1.4.1717) and JASP (version 0.16.1). Descriptive statistics were used to describe medication adherence and percentages for categorical variables. The influence of sex was analyzed using the Mann–Whitney test while the influence of age was analyzed by using the Spearman coefficient correlation. To analyze the influence of the type of device and the NMS, a Student’s *t*-test or Welch’s *t*-test with the significance level set at *p* < 0.01 was used after an examination of normality and equality of variances (the Shapiro–Wilk and Levene test, respectively).

To obtain the database for this study, we submitted a request to Pharmanet- NIHDI. Our request was reviewed by the Drug Practices Evaluation Committee to assess potential privacy risks. We received a favorable opinion on the condition that all data provided were anonymized by Pharmanet. At the time of our request to Pharmanet, it was not necessary to obtain approval from an ethics committee in Belgium for the use of anonymized data in retrospective studies.

## 5. Conclusions

In conclusion, the LABA-LAMA pharmacological class of drugs could be more widely dispensed given their added value compared to the LABA-ICS combination in COPD. Despite some missing information, such as patients who did not initiate treatment or hospital deliveries of medication, our study provides an overview of adherence to targeted drugs at the national level. Medication adherence varied greatly between the different pharmacological classes of inhaled asthma and COPD medications. ICS alone or in combination with LABA had the lowest medication adherence and persistence while these were the highest for the LAMA and LABA-LAMA associations. It would be interesting to carry out further studies to evaluate the influence of other factors than those studied in this study, such as disease severity, comorbidities, non-pharmacological treatments, or lifestyle changes.

Concerning the new medication interview introduced in Belgium in 2013, it seemed to have a positive impact on medication adherence although few patients completed the two interviews. In addition, only a minority of the targeted patients took advantage of this new service. It might be interesting to improve awareness among targeted patients and remind professionals of the inclusion criteria and the objectives of this NMS. The NMS could help to improve medication adherence and health literacy, as well as chronic disease management, preventing complications and disease progression. Since 2018 these interviews for the proper use of medicines were extended to other populations such as patients at risk of developing diabetes. It would be interesting to implement a tool to assess medication adherence in chronic treatments through prescription refills in pharmacy software. This would enable pharmacists to easily detect poorly adherent patients and then encourage the completion of an NMS. This new service allows pharmacists to play a central role in the management of targeted patients and could be extended to other chronic pathologies. It would be of interest to conduct further studies to assess the impact of this new service on medication adherence and in patient care.

## Figures and Tables

**Table 1:** Characteristics of the population studied by pharmacological class.

	ICS	LABA	LAMA	LABA-ICS	LABA-LAMA
Number of patients	521,634	77,747	96,462	741,284	24,694
Sex					
Males (%)	48.8	56.7	63.9	47.5	64.6
Females (%)	51.2	43.3	36.1	52.5	35.4
Age					
Mean (years)	31	64	68	53	68
Median (years)	14	67	69	56	68

ICS = inhaled corticosteroids; LABA = long-acting beta agonists; LAMA = long-acting muscarinic antagonists.

**Table 2:** The three most prescribed molecules by pharmacological class in the studied population.

	ICS	LABA	LAMA	LABA-ICS	LABA-LAMA
1.	Budesonide (54.73%)	Indacaterol (54.22%)	Tiotropium bromide (91.38%)	Salmeterol and fluticasone (38.98%)	Indacaterol and glycopyrronium bromide (76.97%)
2.	Fluticasone (35.65%)	Formoterol (37.21%)	Glycopyrronium bromide (6.37%)	Formoterol and budesonide (36.09%)	Vilanterol and umeclidinium bromide (19.90%)
3.	Beclometasone (9.62%)	Salmeterol (8.57%)	Aclidinium bromide (1.83%)	Formoterol and beclometasone (17.51%)	Olodaterol and tiotropium bromide (2.27%)

**Table 3:** The three physician specialties prescribing the most asthma and COPD medications by pharmacological class.

	ICS	LABA	LAMA	LABA-ICS	LABA-LAMA
1.	GPs (72.0%)	GPs (85.5%)	GPs (83.1%)	GPs (83.5%)	GPs (68.2%)
2.	Pediatricians (20.0%)	Specialists in respiratory medicine (9.1%)	Specialists in respiratory medicine (12.6%)	Specialists in respiratory medicine (10.4%)	Specialists in respiratory medicine (27.2%)
3.	Specialists in respiratory medicine (4.4%)	Pediatricians (1.2%)	Specialists in internal medicine (1.0%)	Pediatricians (1.7%)	Specialists in internal medicine (1.6%)

GPs = general practitioners.

**Table 4:** Medication adherence: CMA without TE.

CMA	ICS	LABA	LAMA	LABA-ICS	LABA-LAMA
Mean ± σ	0.211 ± 0.274	0.692 ± 0.318	0.797 ± 0.236	0.420 ± 0.310	0.872 ± 0.192
25th percentile	0.039	0.421	0.664	0.151	0.806
Median	0.089	0.800	0.895	0.333	0.978
75th percentile	0.247	1.000	0.999	0.637	1.000
% of adherent patients (CMA ≥ 0.8)	7.98	50.01	62.83	17.56	75.66

CMA = continuous multiple-interval measures of medication availability; TE = treatment episodes.

**Table 5:** Influence of sex and type of device on CMA.

CMA	ICS		LABA		LAMA		LABA-ICS		LABA-LAMA	
**Sex**	**F**	**M**	**F**	**M**	**F**	**M**	**F**	**M**	**F**	**M**
Mean	0.219 *	0.202 *	0.664 *	0.714 *	0.787 *	0.802 *	0.402 *	0.440 *	0.867	0.874
Std. deviation	0.281	0.266	0.326	0.311	0.244	0.232	0.306	0.313	0.197	0.189
Median	0.092	0.085	0.740	0.841	0.887	0.899	0.307	0.363	0.978	0.978
% of adherent patients (CMA ≥ 0.8)	8.69	7.24	46.15	52.93	61.09	63.82	16.07	19.18	74.71	76.19
**Device**	**DPI**	**pMDI**	**DPI**	**pMDI**	**DPI**	**pMDI**	**DPI**	**pMDI**	**DPI**	**pMDI**
Number of patients	85,985	200,738	74,468	3451	90,912	19,140	555,151	206,691	23,704	1071
Mean	0.377 *	0.116 *	0.701 *	0.502 *	0.796 *	0.856 *	0.417 *	0.480 *	0.883 *	0.654 *
Std. deviation	0.300	0.170	0.316	0.300	0.236	0.200	0.308	0.323	0.183	0.221
Median	0.282	0.056	0.820	0.441	0.892	0.960	0.334	0.403	0.984	0.612
% of adherent patients (CMA ≥ 0.8)	14.27	1.93	51.34	21.44	62.69	72.06	17.13	23.21	77.94	27.16

CMA = continuous multiple-interval measures of medication availability; F = female; M = male; DPI = dry powder inhaler; pMDI = pressurized metered dose inhaler; * *p* < 0.001.

**Table 6:** Influence of the NMS on CMA.

	No Interview	One or Two Interviews
**ICS**		
Mean CMA ± σ	0.211 * ± 0.274	0.218 * ± 0.264
% of adherent patients (CMA ≥ 0.8)	8.01	7.25
**LABA-ICS**		
Mean CMA ± σ	0.420 * ± 0.310	0.431 * ± 0.314
% of adherent patients (CMA ≥ 0.8)	17.55	18.44

* *p* < 0.001.

**Table 7:** Influence of the number of interviews conducted on CMA.

	Only 1st Interview	1st and 2nd Interviews
**Number of interviews delivered**	76,282	5731
Number of patients	70,949	5284
Female	38,413	2852
Male	32,536	2432
Mean age (years)	45	47
Female	46	49
Male	43	45
Specialties of the main prescribers	GP (80.1%)	GP (79.6%)
	Specialist in respiratory	Specialist in respiratory
	medicine (10.5%)	medicine (13.5%)
	Pediatrician (5.8%)	Pediatrician (7.5%)
ICS		
Mean CMA ± σ	0.213 * ± 0.263	0.244 * ± 0.270
% of adherent patients (CMA ≥ 0.8)	7.40	7.83
LABA-ICS		
Mean CMA ± σ	0.420 * ± 0.313	0.484 * ± 0.311
% of adherent patients (CMA ≥ 0.8)	18.71	22.99

GP = general practitioner; * *p* < 0.001.

**Table 8:** Results of the CMA calculation with the TE.

CMA	ICS	LABA	LAMA	LABA-ICS	LABA-LAMA
	First episode				
Mean ± σ	0.432 * ± 0.356	0.913 * ± 0.178	0.837 * ± 0.200	0.632 * ± 0.283	0.914 ± 0.154
% of adherent patients (CMA ≥ 0.8)	24.42	83.05	68.30	35.34	83.25
Mean episode duration (days)	85	250	461	214	293
	Second episode				
N	358,651	44,784	25,885	527,985	10,631
N with at least two deliveries during episode	85,803	24,459	17,593	217,316	6617
Mean ± σ	0.412 * ± 0.366	0.801 * ± 0.257	0.859 * ± 0.208	0.694 * ± 0.298	0.911 ± 0.153
% CMA ≥ 0.8	24.51	63.86	72.53	37.12	80.66
Mean episode duration (days)	55	175	186	113	218
	Third or more episode				
N	234,471	39,211	14,367	342,039	7014
N with at least two deliveries during episode	75,091	24,807	8265	191,263	4062
Mean ± σ	0.509 * ± 0.389	0.861 * ± 0.230	0.912 * ± 0.160	0.816 * ± 0.249	0.921 * ± 0.151
% CMA ≥ 0.8	35.11	74.34	82.61	65.08	84.59
Mean episode duration (days)	62	153	140	125	168
Mean number of episodes	1.999	2.059	1.466	1.810	1.693

* *p* < 0.001.

## Data Availability

The data presented in this study are available on request from the corresponding author.
